# Cost reduction associated with telemedicine evaluation of low-risk orthopedic assessment: A cost-minimization analysis

**DOI:** 10.1051/sicotj/2026039

**Published:** 2026-07-27

**Authors:** Noel Oizerovici Foni, Daniel Tavares Malheiro, Kauê Capellato Junqueira Parreira, Tarso Augusto Duenhas Accorsi, Flavio Tocci Moreira, Karen Francine Köhler, Carlos Henrique Sartorato Pedrotti, Nelson Wolosker

**Affiliations:** 1 Hospital Israelita Albert Einstein São Paulo Brazil; 2 Economia da Saúde, Hospital Israelita Albert Einstein São Paulo Brazil; 3 Departamento de Telemedicina, Hospital Israelita Albert Einstein São Paulo Brazil

**Keywords:** Telemedicine, Orthopedics, Cost-minimization analysis, Operational costs, Economic impact

## Abstract

*Introduction:* Despite the growing adoption of teleorthopedics, predominantly targeting low-complexity cases, the cost-minimization potential of this approach remains insufficiently characterized. *Methods:* The study employed a cost-minimization analysis to compare telemedicine triage against conventional in-person care for orthopedic patients in a private hospital in São Paulo, Brazil. The analysis focused on cost as the primary outcome, assuming clinical equivalence between interventions. The perspective was hospital-based, considering operational expenses in the emergency department. Data were derived from historical cost records using absorption costing. A decision tree model was developed using TreeAge Pro 2024, and a sensitivity analysis was conducted to evaluate cost variations. Results adhered to CHEERS guidelines and ISPOR economic reporting standards. *Results:* The economic analysis revealed an average cost reduction of 48% (US$ 130.00) associated with teleorthopedics, with sensitivity analysis indicating potential 22 savings ranging from 25% (US$ 72.00) to 54% (US$ 146.00). The financial benefit was most pronounced in clinical pathways without diagnostic testing, with a 0.78 probability of cost savings compared to standard care. The consultation duration and telemedicine operational costs are the most influential factors in the cost–benefit outcome. *Conclusion:* Teleorthopedics demonstrates substantial cost-saving potential, particularly in scenarios without diagnostic testing. Consultation duration and operational costs emerge as key economic drivers.

## Introduction

Telemedicine (TM) represents a transformative approach in healthcare, delivering multimodal medical assessments through the integration of video, audio, and digital imaging to provide timely and cost-effective solutions for the population [1]. TM has demonstrated reliable accuracy in the clinical evaluation of a wide range of conditions, including orthopedic disorders [2, 3].

In orthopedics, TM has consistently demonstrated diagnostic reliability. For example, remote consultations for low-risk conditions, such as ankle sprains, hand or foot contusions, and neck or back pain, have demonstrated diagnostic agreement with in-person specialists and yield high patient satisfaction [2, 3]. Recent studies further confirm that Teleorthopaedics (TO) across various conditions is non-inferior to traditional in-person visits [1, 4]. Overall, TO has proven to be a safe and efficient alternative for delivering orthopedic care, particularly to patients in remote or underserved areas. It maintains clinical accuracy while supporting continuous care in many clinical situations. This approach expands access to care, optimizes clinical follow-up, and improves the overall efficiency of orthopedic care [1].

The potential for cost reduction is a primary driver of TO adoption, alongside the goal of expanding medical access alongside the goal of expanding access. TM may reduce costs through faster interactions, decreased patient travel, improved scalability, enhanced provider efficiency, and task delegation to technological solutions [5, 6]. Often, telehealth serves as a partial substitute for in-person visits, raising the critical question of how much cost reduction this substitution truly represents [7].

Cost considerations play a crucial role in evaluating new digital health strategies [8, 9]. However, due to the wide range of analytical approaches and the heterogeneity of existing studies, the cost-effectiveness of these interventions remains a subject of debate in the literature [10, 11].

In orthopedic practice, TO can be cost-effective by reducing infrastructure requirements and operational overhead. TO may lower costs through faster interactions, decreased patient travel, improved scalability, greater provider efficiency, and by leveraging technology for task delegation. One method for evaluating cost-effectiveness is the cost-minimization analysis, an economic evaluation method used when alternative interventions demonstrate equivalent efficacy and safety. In such scenarios, the analysis focuses solely on comparing the costs associated with each option, aiming to identify the most economically efficient alternative – whether for the healthcare system, patients, or society [6, 12]. This type of evaluation is particularly relevant when clinical outcomes are proven to be similar, allowing decision-making to be based solely on the cost of interventions. Within the context of telehealth, cost-minimization analysis can be useful in choosing between remote and in-person care modalities, provided that clinical equivalence has been established [6].

To date, few studies have comprehensively analyzed the available evidence on cost-minimization related to TO, and to our knowledge, none have synthesized this evidence specifically from the perspective of the Brazilian healthcare system [7].

This study aimed to evaluate and compare the cost-minimization of an initial TO consultation strategy with a traditional in-person care model in a cohort of 101 patients with low-risk orthopedic conditions who achieved similar clinical outcomes.

## Methods

### Study design and population

A total of 101 adult patients were prospectively enrolled (≥18 years) presenting with at least one acute orthopedic complaint (cervical pain, low back pain, extremity contusion, or ankle sprain), who spontaneously sought in-person care at the emergency department. This was a single-center, randomized, non-inferiority trial. All patients provided informed consent to participate in this prospective study, conducted in accordance with the guidelines of the institution’s Research Ethics Committee (CAAE 46603821.9.0000.007) and registered at ClinicalTrials.gov (Identifier: NCT04981002). These data were obtained and analyzed from a previously published study [4].

After enrollment, patients were randomly allocated into two groups: the TO Group (*n* = 50) received a TO consultation with a general practitioner (GP), followed by a subsequent in-person evaluation by an orthopedic specialist. The IP Group (*n* = 51) underwent a direct, face-to-face consultation with an orthopedic specialist. The study compared both approaches using the same cohort to assess the non-inferiority of TO. Eligibility was strictly defined to ensure a consistent ‘low-complexity’ profile. Inclusion criteria were limited to patients presenting with standardized musculoskeletal complaints, specifically: Neck pain, low back pain, ankle sprain, and hand-foot contusion [4]. During this scientific validation phase, a mandatory hybrid protocol was implemented: all patients initially triaged via TO underwent a subsequent, independent in-person evaluation by an orthopedic specialist. This design was chosen to ensure patient safety and to calculate the diagnostic concordance between the two models [4].

### Time horizon and analytical perspective

Given that this was a hospital-based protocol, the time horizon considered was limited to the initial care period during patients’ visits to the emergency unit, specifically within the emergency care department. The analysis adopted the perspective of a private hospital, using actual incurred costs.

### Benefits

The benefit measured in the analysis corresponded to the operational monetary benefit generated by the intervention, primarily through the increased care capacity of the emergency unit. Time saved in face-to-face consultations was monetized based on the unit’s cost per minute of operation.

Since TO care has proven non-inferiority compared to in-person care, as shown in a previously published study [4], the economic benefit was assessed from the provider’s perspective (a private hospital in the city of São Paulo) by evaluating the operational gains generated through the implementation of TO. This model reduces the time spent utilizing limited resources, such as those of an urgent care unit. The valued benefit was the time saved at the emergency unit, specifically during the triage and initial consultation phases. As a result, patients using the TO alternative arrive at the urgent care service with a diagnostic plan already in place, when necessary. This streamlined the use of urgent care resources, allowing more patients to be treated without the need to expand the unit’s capacity.

### Costs

The cost estimation in this study was based on the operational reality of a private hospital in São Paulo, Brazil. Data collection was conducted using the institution’s previously adopted cost accounting records. For each hospitalization, all consumed resources and services were individually allocated to the respective patient. The costing methodology applied was absorption costing, whereby indirect costs are proportionally allocated in the final hospital bill.

To mitigate temporal bias and account for inflationary effects over the study period, all costs were updated based on the hospital’s cost table from the final month of data collection. This standardization ensured greater accuracy in the cost-minimization analysis. Additionally, all monetary values were converted to U.S. dollars (USD) using the purchasing power parity (PPP) average exchange rate for the period between October 1, 2021, and November 30, 2022. The adopted exchange rate was US$1 = R$5.23, according to the Central Bank of Brazil (accessed in June 2024).

The mean cost of in-person consultations was R$726.28 (US$138.87), with an average consultation time of 20 min. For telemedicine consultations, the average cost was R$389.90 (US$74.55), with an average duration of 10 min.

Regarding the cost of care modalities, the mean cost of in-person consultations was R$ 726.28 (US$ 138.87), with an average consultation time of 20 min, resulting in a unit cost of R$ 36.31 (US$ 6.94) per minute. For telemedicine consultations, the average cost was R$ 389.90 (US$ 74.55), with an average duration of 10 min, resulting in a unit cost of R$ 38.99 (US$ 7.45) per minute. These unit costs represent the total institutional burden per minute. The fiscal benefit in the cost-minimization analysis is thus derived from the reduction in total minutes consumed per patient episode in the telemedicine pathway.

For the decision tree model, the following probabilities were adopted. In the in-person consultation group, the probability of undergoing diagnostic testing was 0.73, while the complementary probability (0.27) represented patients who did not undergo any additional testing. In the telemedicine group, 78% of patients (probability = 0.78) required follow-up in-person visits with diagnostic exams, whereas 22% (probability = 0.22) did not undergo any tests following the remote consultation (Table 1).

**Table 1:** Model input parameters: baseline clinical and economic values.

Variable name (unit)	Variable base value
In-person length of stay (h)	20/60
Telemedicine length of stay (h)	10/60
Telemedicine cost (US$)	74.55
Telemedicine exams probability	0.78
In-person cost (US$)	138.87
In-person exams probability	0.73

### Economic modeling

**Figure 1:**
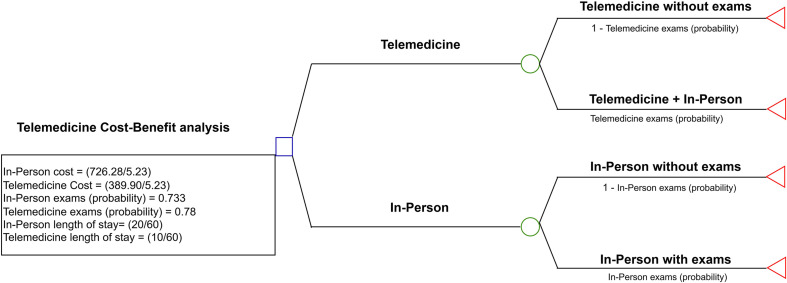
Schematic representation of the decision tree model comparing telemedicine versus in-person orthopedic consultation pathways.

A decision tree model was developed for the analysis based on the consolidated cost and benefit data obtained in the study (Figure 1). This model comprises nodes, branches, and outcomes. The decision node, represented by a square, denotes the primary choice under consideration – in this case, the adoption of a healthcare technology. The decision node is followed by a chance node, represented by a circle, which illustrates the possible subsequent events. Each outcome is associated with its respective probability, reflecting the likelihood of occurrence. The terminal node, represented by a triangle, indicates the health outcome value assigned to a specific result.

The model was constructed using TreeAge Pro Healthcare 2024 software (version 24.2.1-v2024). A probabilistic sensitivity analysis was conducted using 10,000 Monte Carlo simulations to assess the impact of variations in outcomes and costs within predefined ranges (Table 2). Results were presented using a Tornado diagram, which identifies the extent to which each variable, in a univariate manner, influences the variation in expected value (EV).

**Table 2:** Clinical and economic variables: summary of parameters and variation intervals considered in the simulation model.

Variable name	Variable low	Variable base	Variable high
In-person length of stay (h)	0.15	0.33	0.66
Telemedicine length of stay (h)	0.08	0.17	0.33
Telemedicine cost (US$)	57.36	74.55	86.04
Telemedicine exams probability	0.50	0.78	1.00
In-person cost (US$)	114.72	138.87	152.96
In-person exams probability	0.50	0.73	1.00

For the economic modeling, methodological guidelines for economic evaluations as recommended by the Brazilian Ministry of Health were followed. Additionally, the CHEERS checklist was applied to ensure compliance with good reporting practices for health economic evaluations, as outlined by the ISPOR Health Economic Evaluation Publication Guidelines – CHEERS: Good Reporting Practices. The economic modeling followed the methodological guidelines for economic evaluations established by the Brazilian Ministry of Health, and the CHEERS 2022 checklist was applied to ensure compliance with international best practices for reporting economic evaluations, as recommended by the ISPOR Health Economic Evaluation Publication Guidelines – CHEERS: Good Reporting Practices [13].

### Cost–benefit calculation model

The cost analysis was performed using two mathematical expressions designed to estimate the economic efficiency of telemedicine consultations compared to traditional in-person care. The variables used in the formulas are defined as follows:

*C*_ip_: Total in-person cost*.*

*C*_tm_: Total telemedicine cost.

*L*_ip_: In-person length of stay (LOS).

*L*_tm_: Telemedicine length of stay (LOS).

*R*_ip_ = *C*_ip_/*L*_ip_: Cost per unit of time for in-person care.

*R*_tm_ = *C*_tm_/*L*_tm_: Cost per unit of time for telemedicine care.

The formulas are described as follows:

Formula 1:

This equation calculates the savings generated by the difference in cost rates applied to the telemedicine duration, offset by the total in-person baseline:

$$S_{1}=\left(R_{\mathrm{ip}}-R_{\mathrm{tm}}\right)\times L_{\mathrm{tm}}-C_{\mathrm{ip}}.$$

Formula 2:

$$S_{2}=\left(R_{\mathrm{ip}}-R_{\mathrm{tm}}\right)\times \left(L_{\mathrm{ip}}-L_{\mathrm{tm}}\right)-C_{\mathrm{tm}}.$$

This equation determines the savings based on the efficiency gain (difference in LOS) and the differential in cost rates, adjusted for the telemedicine baseline:

These equations generated the financial savings of TO consultations under two scenarios:

- *Scenario 1:* Savings relative to in-person cost.
- *Scenario 2:* Savings relative to telemedicine cost.

**Figure 2:**
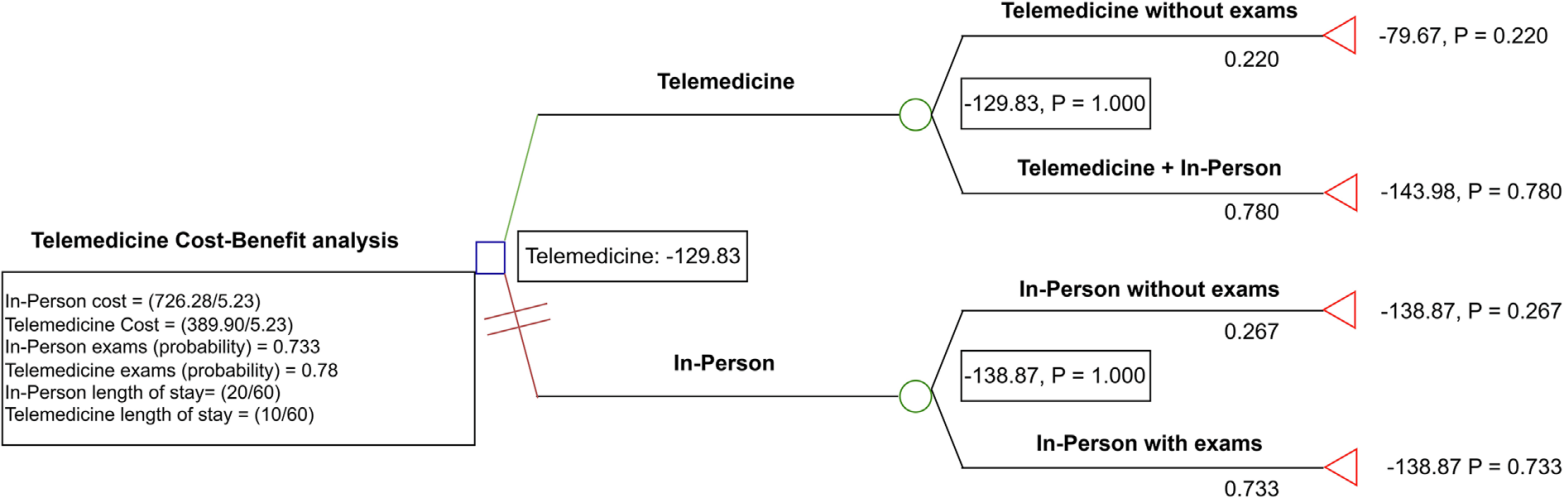
Schematic representation of the decision model, including associated costs, transition probabilities, and terminal nodes.

Baseline values for costs, consultation times, and probabilities were fixed (Figure 2; Table 2). The resulting values represent the estimated cost–benefit of implementing TO in the evaluated clinical context.

To assess the robustness of the economic model, a probabilistic sensitivity analysis was performed using a Tornado diagram. The analysis tested variations in key parameters of the cost–benefit model within predefined ranges (Table 3).

**Table 3:** Summary of sensitivity analysis results: Incremental Cost-Effectiveness Ratios (ICER) and net monetary benefits across different scenarios.

Variable name	Variable low	Variable high	Impact	Low (US$)	High (US$)
In-person length of stay (h)	0.15	0.66	Decrease	−138.87	−64.16
Telemedicine length of stay (h)	0.08	0.33	Increase	−138.87	−74.56
Telemedicine cost (US$)	57.36	86.04	Decrease	−138.87	−109.23
Telemedicine exams probability	0.50	1.00	Decrease	−138.87	−112.06
In-person cost (US$)	114.72	152.96	Decrease	−134.13	−114.72
In-person exams probability	0.50	1.00	Increase	−130.12	−130.12

Each variable was individually varied while keeping others constant to measure its isolated impact on the expected value (EV). This approach aimed to identify which factors most influenced cost variations and to simulate plausible real-world scenarios. For example, although the base case assumed a 0.78 probability of diagnostic test requests during telemedicine consultations, this rate was varied up to 1.0 to reflect contexts where testing may be universal.

## Results

The economic model demonstrated a significant financial advantage of telemedicine (TM) consultations compared to traditional in-person (IP) visits. In the base-case scenario, the transition to TM resulted in an expected cost saving of US$ 130.12 per consultation.

**Figure 3:**
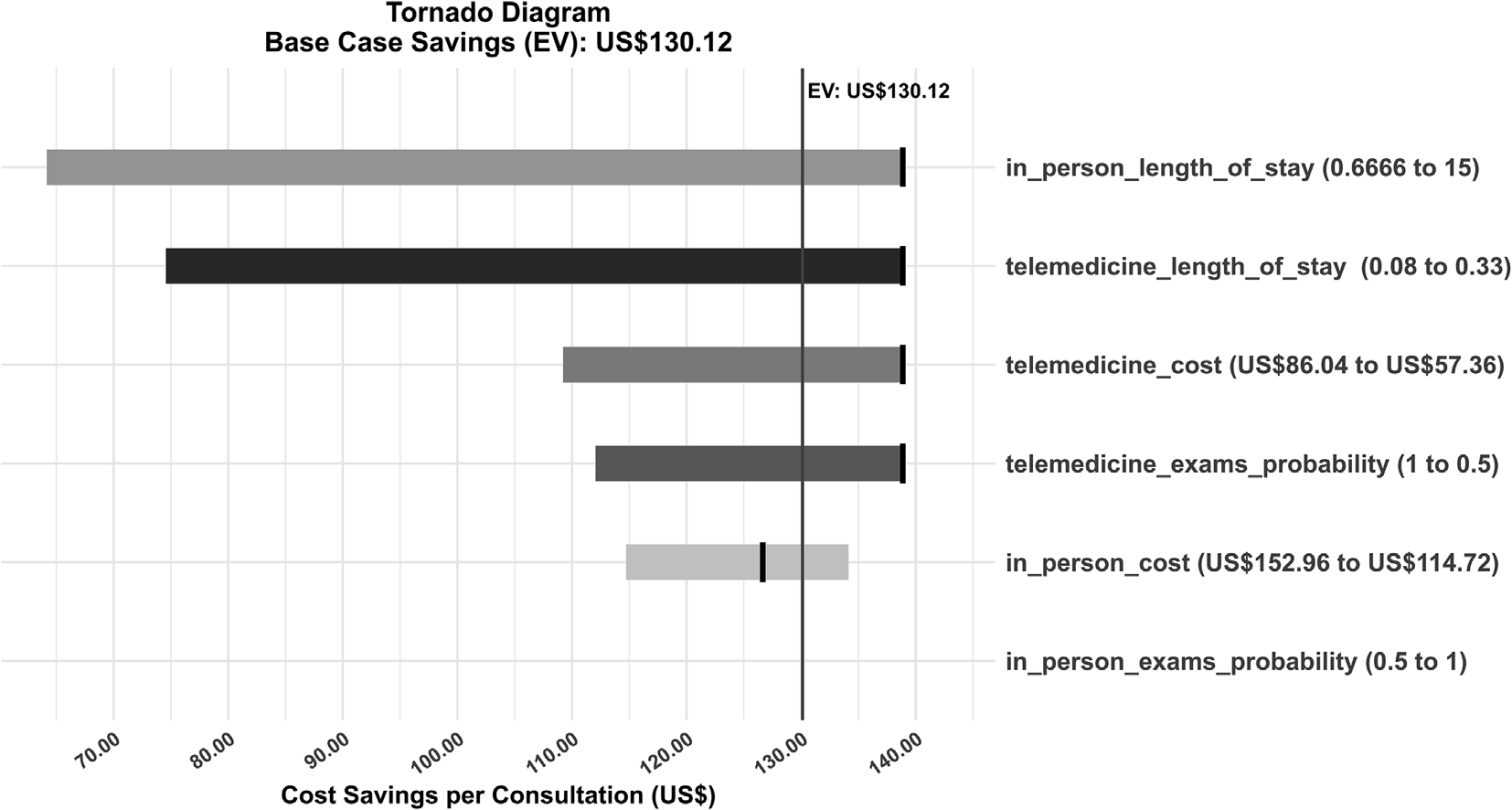
Tornado diagram representing the one-way sensitivity analysis of cost savings per consultation.

To account for parameter uncertainty, a one-way sensitivity analysis was performed. The results indicated that the financial benefit of TM remains robust, with savings ranging from US$ 64.16 to US$ 138.54 across all tested variations (Figure 3).

The Tornado diagram identifies the key drivers of this economic outcome. The duration (length of stay) of both in-person and telemedicine consultations exerted the most substantial influence on cost variation. Specifically, as the duration of in-person visits increased, the relative savings offered by TM became even more pronounced. In contrast, factors such as the absolute cost of consultations and the probability of diagnostic test referrals had a comparatively lower impact on the overall economic benefit.

## Discussion

This study evaluated the implementation of a TO triage process for orthopedic patients with low-complexity musculoskeletal complaints, compared with the standard of care without telemedicine. As both arms assessed the same population, no adjustment or balancing was necessary.

Although clinical assessment in orthopedics has traditionally relied on comprehensive physical maneuvers, the field has a long-standing history of implementing simplified, objective protocols to streamline care. Since the landmark development of the Ottawa Ankle Rules in 1992 [14], which utilized specific physical findings to determine the necessity of radiographic imaging, orthopedics has continuously integrated innovations to refine diagnostic accuracy. While it is acknowledged that seemingly low-risk cases may harbor underlying complexities, the evolution of validated clinical rules suggests that standardized assessment models can safely guide initial management. In this context, telemedicine emerges as a contemporary extension of this trend, offering a digital framework to complement traditional practice without compromising the rigor established by classical semiology.

The economic analysis presented in this study reinforces the growing body of evidence supporting the cost-minimization of TO consultations, particularly for low-complexity musculoskeletal conditions. Our findings align with recent research indicating that telemedicine can lead to substantial cost savings without compromising clinical outcomes [15, 16].

A randomized controlled trial conducted in Northern Norway demonstrated that video-assisted orthopedic consultations in remote clinics were cost-effective compared to standard outpatient consultations at specialist hospitals, provided that the number of consultations exceeded 151 per year [2]. This threshold highlights the significance of patient volume in achieving the economic benefits of telemedicine implementations. Previous studies have already established that clinical outcomes and patient safety in TO are non-inferior to traditional in-person consultations [2, 3]. General practitioners achieved a 98.0% agreement rate (95% CI: 89.6%–100.0%) when comparing TM assessments to in-person clinical findings. The only exception involved one patient (2.0%) whose condition was categorized as a foot injury via TM but was ultimately confirmed to be an ankle sprain in person [4].

Further supporting these findings, a study analyzing telemedicine in orthopedic oncology reported significant cost savings for patients and healthcare systems, primarily due to reduced travel and associated expenses, without negatively impacting clinical outcomes [17]. Moreover, the integration of telemedicine in orthopedic sports medicine practices has been associated with decreased costs and wait times, alongside increased patient satisfaction [18]. Notably, the COVID-19 pandemic accelerated the adoption of telehealth services across various medical specialties, highlighting its value in ensuring care.

This study provides empirical evidence of the cost-saving potential of TO within the Brazilian healthcare context. The observed average cost reduction of 48% per patient encounter suggests the potential economic viability within the specific context of this institutional mode, the economic viability of telemedicine as a sustainable alternative to traditional in-person care, particularly in scenarios where diagnostic testing is not required. It is important to note that the cost-minimization of telemedicine is influenced by several factors, including consultation duration, operational costs, and the frequency of diagnostic test utilization [15]. Sensitivity analyses in our study further revealed that these variables significantly impact the overall cost–benefit outcome, emphasizing the need for careful consideration in the implementation and scaling of TO services.

While our findings are promising, further research is warranted to investigate the long-term economic impacts of TO services, including their effects on healthcare utilization patterns, patient adherence to treatment plans, and the overall efficiency of the healthcare system.

This study has several limitations that must be acknowledged. First, it was conducted at a single institution, which may limit the generalizability (external validity) of our findings to other healthcare settings or regions with different operational structures. Second, the sample size is relatively small, representing a pilot evaluation of the TO triage process. Third, the economic evaluation is based on secondary modeling using observed time and cost estimates, rather than a primary longitudinal accounting of all expenditures.

Additionally, a specific limitation is the difference in professional qualifications between the study groups at the initial assessment. While the TO group was triaged by GPs, the IP group underwent direct evaluation by orthopedic specialists. Future studies should aim for a “like-for-like” comparison to isolate the technological impact on costs. Finally, our model does not capture all real-world clinical complexities, such as telemedicine platform maintenance, digital literacy barriers, or potential long-term “downstream” costs. Future multi-center studies with larger cohorts are necessary to confirm these preliminary economic benefits.

Beyond institutional settings, studies examining the integration of TO into public healthcare systems, particularly in low- and middle-income countries, would provide valuable insights into the scalability, sustainability, and equity of digital health. Such research is essential to determine if the cost-saving benefits observed in this pilot can be replicated in resource-constrained environments where the burden of musculoskeletal disease is high.

TO offers significant potential for cost reduction, especially when diagnostic tests are not required. This finding highlights the efficiency of remote consultations in managing low-complexity musculoskeletal conditions, where clinical evaluation alone is often sufficient for diagnosis and treatment. By reducing the need for face-to-face interactions and avoiding unnecessary ancillary testing, tele-orthopedics can alleviate the operational burden on emergency departments and improve care delivery without compromising clinical quality.

Moreover, the time efficiency gained through shorter teleconsultations translates into increased patient throughput and better resource utilization, particularly in high-volume or resource-limited settings. These cost savings are not only beneficial to healthcare providers and systems but may also improve patient access to timely orthopedic evaluation, especially in underserved or geographically remote areas. As health systems worldwide seek to expand digital health infrastructure, TO triage represents a potential model for improving the value of care, although its scalability requires further validation in broader clinical settings. Future research should focus on assessing long-term outcomes, implementing effective strategies, and exploring the broader implications of integrating TO into both private and public healthcare networks.

## Conclusion

This single-institutional study suggests that teleorthopedics (TO) consultations may be economically efficient compared with in-person care, specifically when applied to selected low-complexity musculoskeletal cases. A decision tree model and Monte Carlo simulations provided support for the robustness of these findings within the defined parameters. However, the economic benefits are context-dependent and rely on specific conditions, such as consultation duration, operational costs, and the probability of diagnostic test referrals. While TO presents a potential for cost reduction, clinical caution remains essential when triaging low-risk conditions, and further research is needed to validate these findings across broader clinical settings.

## Data Availability

The datasets generated during and/or analyzed during the current study are available from the corresponding author on reasonable request.
